# Computer Tomography Radiomics-Based Nomogram in the Survival Prediction for Brain Metastases From Non-Small Cell Lung Cancer Underwent Whole Brain Radiotherapy

**DOI:** 10.3389/fonc.2020.610691

**Published:** 2021-02-11

**Authors:** Ji Zhang, Juebin Jin, Yao Ai, Kecheng Zhu, Chengjian Xiao, Congying Xie, Xiance Jin

**Affiliations:** ^1^ Department of Radiotherapy Center, The First Affiliated Hospital of Wenzhou Medical University, Wenzhou, China; ^2^ Department of Radiation and Medical Oncology, The Second Affiliated Hospital of Wenzhou Medical University, Wenzhou, China

**Keywords:** brain metastasis, non-small cell lung cancer, whole brain radiotherapy, overall survival, radiomics, nomogram

## Abstract

Prognostic parameters and models were believed to be helpful in improving the treatment outcome for patients with brain metastasis (BM). The purpose of this study was to investigate the feasibility of computer tomography (CT) radiomics based nomogram to predict the survival of patients with BM from non-small cell lung cancer (NSCLC) treated with whole brain radiotherapy (WBRT). A total of 195 patients with BM from NSCLC who underwent WBRT from January 2012 to December 2016 were retrospectively reviewed. Radiomics features were extracted and selected from pretherapeutic CT images with least absolute shrinkage and selection operator (LASSO) regression. A nomogram was developed and evaluated by integrating radiomics features and clinical factors to predict the survival of individual patient. Five radiomics features were screened out from 105 radiomics features according to the LASSO Cox regression. According to the optimal cutoff value of radiomics score (Rad-score), patients were stratified into low-risk (Rad-score <= −0.14) and high-risk (Rad-score > −0.14) groups. Multivariable analysis indicated that sex, karnofsky performance score (KPS) and Rad-score were independent predictors for overall survival (OS). The concordance index (C-index) of the nomogram in the training cohort and validation cohort was 0.726 and 0.660, respectively. An area under curve (AUC) of 0.786 and 0.788 was achieved for the short-term and long-term survival prediction, respectively. In conclusion, the nomogram based on radiomics features from CT images and clinical factors was feasible to predict the OS of BM patients from NSCLC who underwent WBRT.

## Introduction

Brain metastasis (BM) is the most frequent intracranial malignancy and remains a leading cause of morbidity and mortality in both men and woman despite advances in surgical, systemic, and radiotherapy treatments (1). Lung cancer is the most common primary origin for patients with BM, where non-small cell lung cancer (NSCLC) accounts for approximately 80% of all lung cancers (2). The prognosis of NSCLC patients with BM has significantly worsened with the median overall survival (OS) varying from 2.8 to 25.3 months (3). Studies indicated that the prognosis of individual BM patients may be affected by a few clinical factors, such as the type of primary cancer, systemic control, treatment modality, treatment response, *etc* (4, 5). The identification of these prognostic factors before or early after the beginning of treatment was believed to be helpful in improving the treatment outcome for patients with BM by adjusting and choosing the right management strategy (6).

In the past decades, several prognostic models had been suggested to predict the survival of BM patients (7–9). Recently, prognostic models such as Golden Grading System (GGS), Disease-Specific Graded Prognostic Assessment (DS-GPA), Score Index for Radiosurgery (SIR) in brain metastases, *etc.* have been published (10–12). Although these models and the suggested prognostic factors have facilitated the prediction of survival, lack of individualized survival probability and disproportional size of prognostic groups observed in these models hindered their wide application for clinical use (9). Biomarkers derived from genomic and proteomic data in primary cancers had also been reported to stratify patients into different diagnostic/prognostic groups and lead to more effective treatment paths (13, 14). However, the procedures of acquiring genomic and proteomic biomarkers are usually invasive and are not always technically feasible (15). Studies also pointed out core biopsy specimens may not represent the entirety of the tumor due to the spatial heterogeneity of tumors (16, 17).

As an emerging quantitative analysis technique, radiomics has been used to provide valuable information from medical images pertaining to tumor phenotype and microenvironment, which had been used for cancer diagnosis, treatment response monitoring, and outcome prediction for various cancers (18). Recently, Della et al. demonstrated that three-dimensional (3D) quantitative tissue enhancement in pre-treatment cranial magnetic resonance imaging (MRI) may be a radiomic marker to predict the survival of patients with singular BM treated with stereotactic radiation therapy (SRT) (19). Karami et al. also investigated that feasibility of using quantitative MRI (qMRI) biomarkers to predict the outcome of local failure for BM patients treated with SRT (20). Huang et al. pointed out that radiomic features from T1 MRI could potentially be used as surrogate biomarkers for tumor prognosis prediction following gamma knife radiosurgery (GKRS) (21).

The management options for patients with BM have been diverse in the present era, including whole brain radiation therapy (WBRT), hypo-fractionated SRT, stereotactic radiosurgery (SRS), surgical resection, and systemic therapy (22). WBRT is a standard treatment modality for NSCLC patients with multiple BM and remains the cornerstone of management of BM for many years (23). Despite the availability of diverse scoring systems, there is still a lack of consensus regarding the prognostic factors that can help the treatment decision-making concerning the use of WBRT in NSCLC patients with BM (24). On the other hand, although MRI is a more sensitive than computer tomography (CT) for BM detection, contrast enhanced CT (CECT) has been recommended on equal footing with MRI in the 2007 evidence-based ACCP guidelines for the detection of asymptomatic NSCLC metastases (25), as no improvement in survival has been reported based on screening with MRI versus CT (26). CT is also a standard modality in the radiation treatment planning for BM. So the purpose of this study is to investigate the feasibility and sensitivity of CT radiomics based nomogram to predict the survival of patients with BM from NSCLC treated with WBRT.

## Materials and Methods

### Patients and Computer Tomography Acquisition

Patients with BM treated in our institute from January 2012 to December 2016 were retrospectively reviewed in this study. The including criteria were 1) BM metastasized from original NSCLC; 2) BM treated with WBRT; 3) The number of metastases is less than ten; 4) Patients with pretherapeutic CECT images. BM metastasized from other origins and treated with other radiotherapy techniques was excluded. The Ethics Committee in Clinical Research of our institute approved this retrospective study and waived the need of written informed consent (ECCR#2019059). The study was conducted according to the Declaration of Helsinki with confirmation of patient data confidentiality.

BM patients were immobilized with a thermal plastic in supine position for radiotherapy. Cerebral CECT images were acquired using a 16-detector row CT simulator (Brilliance, Phillips, Cleveland OH, USA). The scanning parameters were set identical for these patients at 100 kV, 180–280 mA and a field of view of 450 mm with a 3 mm reconstructed section thickness. Before the CT scan, 100 ml of iodinated contrast material was injected into vein *via* a high pressure injector at a rate of 3.0 to 4.0 ml/s.

### Radiomics Feature Extraction

No preprocessing or normalization was performed for the DICOM CT images. The tumors were manually contoured by a junior radiation oncologist and verified by a senior radiation oncologist *via* a 3D Slicer software (version 4.2.1, https://www.slicer.org). For patients with two or more metastases, all the metastatic tumors were contoured regarding as an individual tumor.

CECT images with contoured target volumes were then imported into python3.0(https://www.python.org). An in-house algorithm in Python was coded to extract texture features automatically from each segmented region of interest (ROI) using Python package (PyRadiomics). A total of 105 radiomics features were extracted from individual BM lesion quantifying phenotypic differences on the basis of shape (*n* = 13), first-order (*n* = 18), and texture (*n* = 74) features. Detailed information on the feature extraction algorithms was shown in **Supplementary Material 1**.

### Feature Selection and Radiomics Signature

For individual patient, radiomics features were analyzed based on the sum of the radiomics values of each lesion divided by the number of lesions. The least absolute shrinkage and selection operator (LASSO) is a computationally attractive alternative to standard covariance selection for sparse high-dimensional graphs and an effective approach for the biomarker selection of high-dimensional data. The LASSO Cox regression model was used to select the effective prognostic radiomics features. Depending on the regulation weight *λ*, LASSO shrinks all regression coefficients towards zero and sets the coefficients of many irrelevant features exactly to zero. A radiomics signature was generated *via* a linear combination of selected features weighted by their respective coefficients.

### Survival Assessment and Nomogram

Kaplan–Meier survival analysis was applied to assess the association between radiomics signature and survival. Patients were divided into high-risk and low-risk groups based on a threshold of the radiomics score (Rad-score). The threshold was estimated based on the training cohort using an optimal cut-point analysis with X-tile software (version 3.6.1, Yale University School of Medicine, New Haven, Conn), and tested on the validation cohort. A weighted log-rank test (G-rho rank test, rho = 1) was used to test the difference between the high-risk and low-risk groups.

Clinical factors that associated with OS were also investigated with univariate analysis and multivariate Cox regression analysis. Factors with a *p* value less than 0.1 in the univariate analysis were included in the multivariate analysis. A nomogram was developed by integrating radiomics features and clinical factors to evaluate quantitatively the survival of individual patient. The performance of radiomics signature and nomogram was evaluated with Harrell concordance index (C-index) (1 indicates perfect concordance; 0.5 indicates no better concordance than chance).

### Statistical Analysis

The OS was defined as the time from the date of first WBRT until death or the last follow-up. Patients were randomly divided into training data set (70%) and validation data set (30%). Categorical variables were compared using the x^2^ test or Fisher exact test. Continuous variables were compared by using the Student *t* test or Mann–Whitney *U* test, when appropriate. Selection of radiomics features and logistic regression model building were done using the “glmnet” package. The receiver operating characteristics (ROC) curve was performed using “pROC” package. The nomogram was achieved using “rms” and “survival” packages. The statistical analyses were conducted with R software (version 3.0.1, http://www.R-project.org), SPSS software (version 19.0, IBM, Armonk, NY, USA) and Origin 2018. For all tests, p < 0.05 was considered as statically significant.

## Results

### Patients’ Characteristics

**Figure 1:**
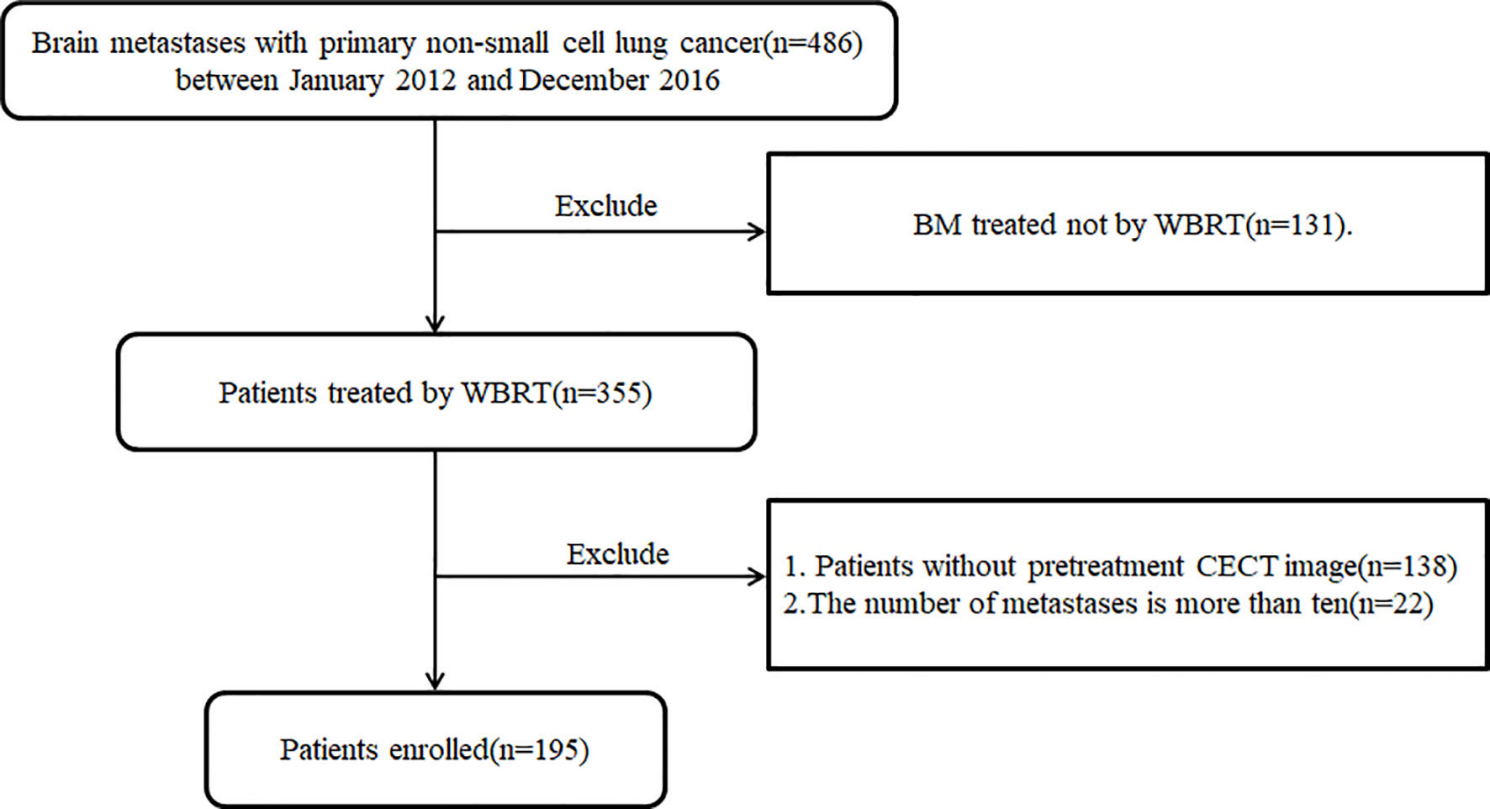
The flowchart of patients’ selection for this retrospective study.

A total of 195 patients (male 132, female 63) with BM from lung cancer were enrolled in this retrospective study, as shown in the flowchart for patient selection in **Figure 1**. Patients were divided into training (133) and validation (62) cohorts with a median and mean age of 62.0, 61.9 years, and 63.0, 63.0 years, respectively. The median and mean OS were 8.7, 13.5 months and 8.8, 12.6 month for the training and validation data sets, respectively. The clinical characteristics were well balanced between the training and validation data sets, as shown in **Table 1**.

**Table 1:** Characteristics of patients in the training and validation cohorts.

Characteristic	Training cohort(N = 133)	Validation cohort(N = 62)	*P* value
Sex, No. (%)			0.99
Male	90(67.7%)	42(67.7%)	
Female	43(32.3%)	20(32.3%)	
Age			0.85
Range	35-88	40-83	
Median	63.0	61.5	
Mean	61.87	61.82	
Karnofsky performance score			0.73
Median	70	70	
No. of metastases lesions, No. (%)			0.38
single	62(46.6%)	24(38.7%)	
multiple	71(53.4%)	38(61.3%)	
Extracranial metastasis, No. (%)			0.92
Yes	77(57.9%)	29(53.2%)	
No	56(42.1%)	23(46.8%)	
Smoking, No. (%)			0.94
Yes	62(46.6%)	30(48.4%)	
No	71(53.4%)	32(51.6%)	
Hypertension, No. (%)			0.019
Yes	52(39.1%)	13(21.0%)	
No	81(60.9%)	49(79.0%)	
Glycuresis, No. (%)			1.00
Yes	20(15.0%)	10(16.1%)	
No	113(85.0%)	52(83.9%)	
Overall Survival(d)			0.56
Range	4-1778	16-1765	
Median	260	247.5	
Mean	403.17	455.29	

### Feature Selection and Radiomics Signature

**Figure 2:**
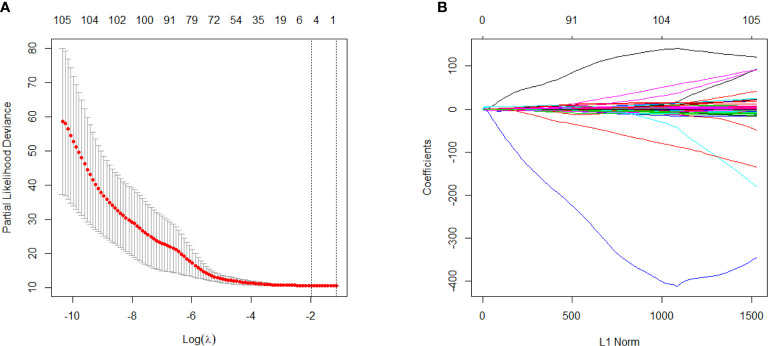
Selection of survival associated radiomics features using the elastic net method, **(A)** Tuning parameter (*λ*) in the elastic net used 10-fold cross-validation *via* maximum area under curve and criterion of minimum standard deviation were followed; **(B)** The coefficient profiles of 105 radiomics features against the L1 norm (inverse proportional to log *λ*).

As shown in **Figure 2**, 10-fold cross validation was performed in the elastic net to tune parameter *λ*, so as to select the radiomics features that were associated with OS. Five radiomics features were screened out from 105 radiomics features according to the LASSO Cox regression analysis. They were one first order feature, two gray-level run length matrix (GLRLM) features, one gray-level size zone matrix (GLSZM) feature and one gray-level different matrix (GLDM) feature. The radiomics signature was constructed based on the Rad-score. Rad-score of individual patients was computed through a linear combination of the selected features weighted by their respective coefficients, as shown in the **Supplementary Data File 2**.

**Figure 3:**
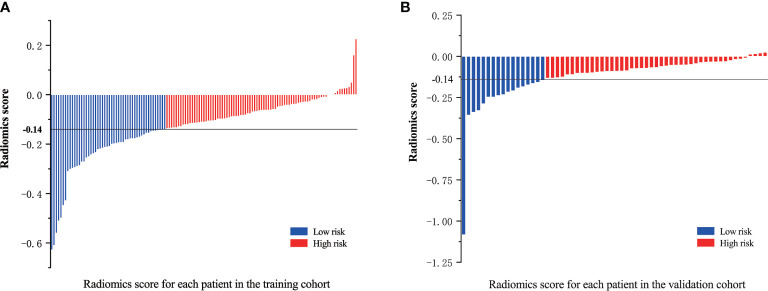
Radiomics scores for each patient in the **(A)** training cohort and **(B)** validation cohort; patients were classified into high and low risk groups with a threshold of −0.14.

**Figure 4:**
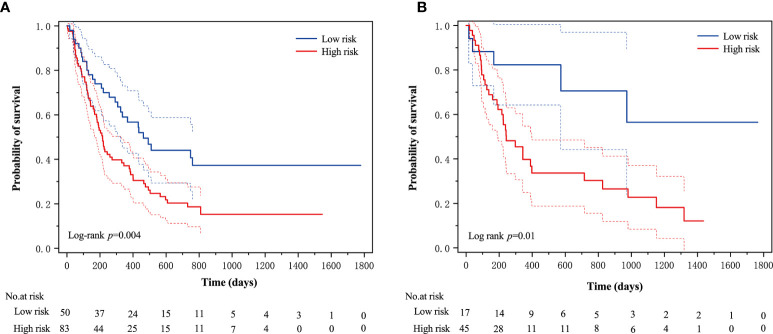
The Kaplan–Meier survival curves of the low- and high-risk groups according to the log-rank test for patients in **(A)** training cohort and **(B)** validation cohort. The vertical dashed line is 95% confidence interval.

An optimal cutoff value of −0.14 was calculated by the X-tile plot based the Rad-scores of patients in the training cohort. The patients were then stratified into low-risk (Rad-score < =−0.14) and high-risk (Rad-score > −0.14) groups. **Figures 3A, B** shows the distribution of Rad-scores in the training cohort and the validation cohort for patients with low and high risk, respectively. Significant survival differences were observed between patients of low and high-risk groups according to the log-rank test, as shown in **Figure 4**. The performance of selected individual radiomics features and the radiomics signature in the prediction of low and high-risk patients was shown in **Table 2**. A C-index of 0.635 was achieved with radiomics score.

**Table 2:** The performance of the selected radiomics features and constructed radiomics signatures.

Radiomics features	Training cohort			Validation cohort		
	C-index	95%CI	*P* value	C-index	95%CI	*P* value
Firstorder_Skewness	0.547	(0.486,0.608)	0.14	0.541	(0.436,0.646)	0.45
GLRLM_LowGrayLevelRunEmphasis	0.546	(0.487,0.605)	0.13	0.570	(0.468,0.679)	0.18
GLRLM_ShortRunLowGrayLevelEmphasis	0.549	(0.489,0.609)	0.11	0.582	(0.482,0.682)	0.11
GLSZM_GrayLevelNonUniformity	0.522	(0.455,0.589)	0.51	0.589	(0.496,0.682)	0.060
GLDM_DependenceNonUniformityNormalized	0.534	(0.471,0.597)	0.29	0.584	(0.478,0.690)	0.77
Radiomics score	0.581	(0.523,0.639)	0.0059*	0.635	(0.536,0.734)	0.0074*

*Statistical significance.

### Risk Factors and Nomogram

**Table 3** shows the results of univariate and multivariate Cox analysis of the risk factors associated with OS in the training cohort. According to the multivariable analysis, sex (HR = 1.733; 95% CI: 1.125–2.794; *p* = 0.014), karnofsky performance score (KPS) (HR = 3.204; 95% CI: 2.003–5.125; *p* < 0.001) and Rad-score (HR = 10.866; 95%CI: 1.711–68.981; *p* = 0.011) were independent predictors for OS.

**Table 3:** Univariate and multivariate Cox regression analysis for risk factors associated with overall survival.

Variable	Univariate analysis			Multivariable analysis		
	HR	95%CI	*p* value	HR	95%CI	*p* value
**Sex**	1.656	(1.059,2.590)	0.027	1.733	(1.125,2.794)	0.014*
**Age (<=56, >56)**	0.991	(0.662,1.484)	0.97			
**KPS (<=70, >70)**	3.450	(2.185,5.447)	<0.001	3.204	(2.003,5.125)	<0.001*
**Extracranial met**	0.514	(0.338,0.783)	0.002	0.682	(0.441,1.053)	0.084
**Smoking**	0.778	(0.520,1.165)	0.22			
**Hypertension**	1.052	(0.695,1.594)	0.81			
**Glycuresis**	0.771	(0.443,1.341)	0.36			
**Rad-score**	14.006	(2.233,87.845)	0.005	10.866	(1.711,68.981)	0.011*

KPS, Karnofsky performance score; met, metastasis; Rad-score, radiomics score.

*Statistical significance.

**Figure 5:**
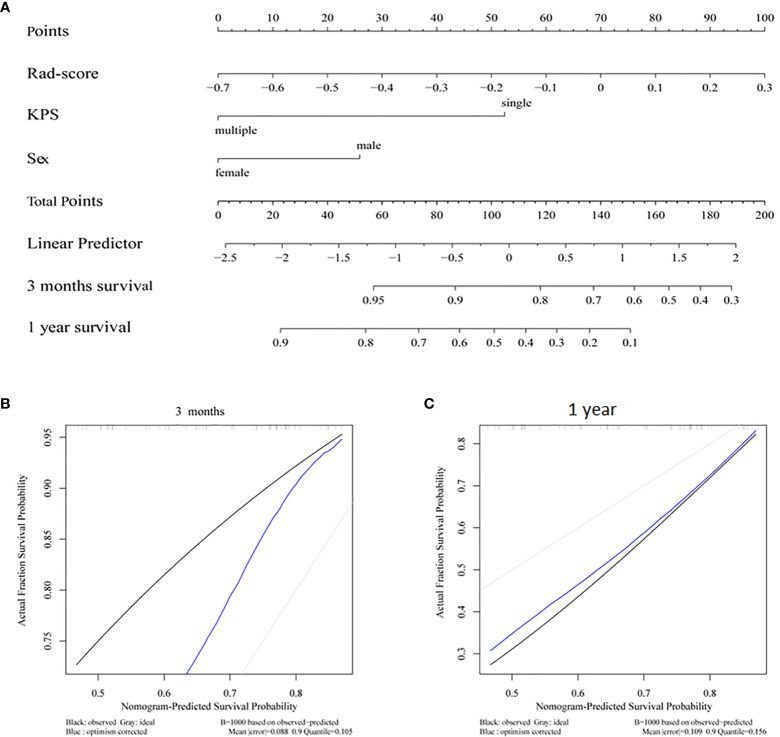
**(A)** Radiomics nomogram integrated with radiomics signature and clinical factor; calibration curves of the radiomics nomogram for **(B)** 3 months survival and **(C)** 1 year survival.

**Figure 6:**
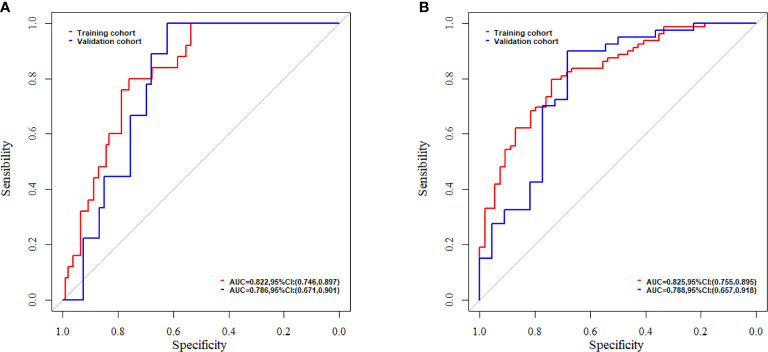
The evaluation of nomogram with receiver operating characteristic curves for **(A)** short-term survival prediction model; **(B)** long-term survival prediction model.

A nomogram was constructed by integrating the clinical factors and radiomics signature (**Figure 5A**) to predict the probability of 3-month OS and 1-year OS after treatment for patients with BM. **Figures 5B, C** demonstrated the calibration curves for the evaluation of agreement between nomogram prediction and actual observation for 3-month and 1-year OS with validation data set, respectively. The C-index of nomogram in training cohort and validation cohort were 0.726, 0.660, respectively. Further verification with ROC was shown in **Figure 6**. An AUC of 0.786 (95% CI: 0.671–0.901) and 0.788(95% CI: 0.657–0.918) was achieved for the short-term and long-term survival prediction, respectively.

## Discussion and Conclusions

In this study, the feasibility and sensitivity of a CT based radiomics nomogram were investigated in the prediction of 3-month and 1-year survival for patients with BM from NSCLC. Sex, KPS and Rad-score were associated with the OS and integrated into the nomogram. A C-index of 0.660 was achieved by the nomogram in survival prediction for BM patients from NSCLC. An AUC of 0.786 and 0.788 was achieved for the 3-month and 1-year survival prediction, respectively.

More than 20% of patients with NSCLC are affected by BM and are associated with poor prognosis (2, 3). Patients with BM were usually reported with remarkable heterogeneity. BM patients may have one or dozens of metastases with varied response or resistance to radiation therapy or chemotherapy (27). Similarly, in this study, there were about 55% (109/195) patients with more than one metastasis with a mean OS around 13 months. Due to this heterogeneity, prognostic features and treatment options for patients with BM should be carefully investigated on an individual basis. Although there is still controversy for the palliative treatment for BM patients with poor prognosis, multidisciplinary palliative therapy must be administered to increase the OS rates of patients with good prognosis (28).

The prediction of survival for BM is usually difficult due to a plethora of factors associated with survival. In the literature, multiple factors, such as control of primary disease, number of metastasis, KPS, age, tumor volume, presence of extracranial metastases *etc.*, were investigated for the prediction of survival of patients with BM (4). The type of treatment is certainly a significant prognostic factor for patients with BM. Whole brain volume reduction and neurocognitive function decline were the major concerns with WBRT (29), while with the development of hippocampal-sparing technique, neurocognitive function and patient-reported symptoms were improved (30). In this study, sex and KPS were correlated with the OS with a HR of 1.733 and 3.204, respectively, according to the multivariate Cox analysis for BM patients treated with WBRT. This is consistent with previously reported models (4, 8, 9).

In this study, radiomics features extracted from CT images were also closely associated with the survival of BM patients as it demonstrated that the HR of Rad-score was 10.866 with a p value of 0.011. Similarly, Huang et al. demonstrated that radiomics features extracted from pre-treatment T1 MRI was an independent prognostic factor of local control for BM patients who underwent gamma knife radiosurgery (21). Karami et al. found it was possible to use MRI based radiomics features to predict local failure early for BM patients treated with SRT (20). Huang et al. also reported that the radiomics features extracted from chest CT images were independent of clinical-pathologic risk factors and significantly correlated with the disease free survival of patients with early-stage NSCLC (31).

With radiomics features alone, a C-index of 0.635 was achieved in the prediction of survival of BM patients who underwent WBRT in this study. This was better than a nomogram integrating clinical factors of primary site, histology, status of primary disease, metastatic spread, age, KPS, and number of brain lesions by Barnholtz-Sloan et al., in which a C-index of 0.60 was reported in the prediction of survival of 2,350 BM patients from seven Radiation Therapy Oncology Group (RTOG) randomized trials (32). Pietrantonio et al. developed a survival prediction nomogram for BM from colorectal cancer and achieved a similar C-index of 0.64 and external validation C-index of 0.73 with the integrating of age, KPS, site of BM and number of BM (33).

With the integrating of radiomics features and clinical factors, the nomogram constructed in this study achieved a C-index of 0.726 and 0.660 for the training and validation cohorts, respectively. This was close to the reported C-index of 0.74 in a study of Park et al. in which the survival prediction of BM patients from hepatocellular carcinoma treated with WBRT with or without resection/radiosurgery was investigated (34). The AUC of our radiomics based nomogram for short-term and long-term survival was 0.786 and 0.788, respectively. These were better than the reported two nomograms for the prediction of early death (<3 months) and long-term survival (>1 year), which was investigated by Zindler et al. with an AUC of 0.70 and 0.67 for early dearth and long-term survival, respectively, for BM patients from NSCLC treated with SRS (35).

Some limitations of the current study are its retrospective design and the risk of selection bias. The nomogram was built and validated internally with data from our institution only; external validations with additional independent data are needed to further evaluate the performance of this nomogram. BM patients from primary sites other than NSCLC were not included in this study, such as breast, colorectal cancer, *etc.* With the development of medical imaging technologies, immobilization methods, and radiotherapy techniques, more and more BM patients were treated with SRT and SRS. A more comprehensive nomogram for patients treated with other than WBRT and based on other image modality radiomics will greatly improve our survival prediction ability and guide tailored treatment for patients with BM.

In conclusion, a nomogram based on radiomics features from CT images and clinical factors was constructed to predict the OS for patients with BM from NSCLC who underwent WBRT. The predicted short-term and long-term survival of BM patients who underwent WBRT will help to adjust and choose the right management strategy, so as to improve the outcome for these patients.

## Data Availability Statement

The original contributions presented in the study are included in the article/**Supplementary Material**; further inquiries can be directed to the corresponding author.

## Ethics Statement

The studies involving human participants were reviewed and approved by The Ethics Committee in Clinical Research of the 1st Affiliated Hospital of Wenzhou Medical University. The informed consent from patients/participants was waived by the Ethics Committee for this retrospective study.

## Author Contributions

JZ, YA, and XJ conceived and designed the study. JZ, KM, and ChX acquired and managed the patients. JZ and JJ analyzed and interpreted the data (e.g., statistical analysis, biostatistics, and computational analysis). JZ, XJ, and CoX wrote, reviewed, and/or revised the manuscript. All authors contributed to the article and approved the submitted version.

## Funding

This work was partially funded by the Wenzhou Municipal Science and Technology Bureau (Nos. 2018ZY016 and H20180003) and National Natural Science Foundation of China under Grant No. 11675122.

## Conflict of Interest

The authors declare that the research was conducted in the absence of any commercial or financial relationships that could be construed as a potential conflict of interest.

## Abbreviations

NSCLC, non-small cell lung cancer; BM, brain metastases; WBRT, whole brain radiotherapy; LASSO, least absolute shrinkage and selection operator; CECT, contrast enhanced computer tomography; OS, overall survival; AUC, area under curve; MRI, magnetic resonance imaging.
