# Supplementary material for: Computer Tomography Radiomics-Based Nomogram in the Survival Prediction for Brain Metastases From Non-Small Cell Lung Cancer Underwent Whole Brain Radiotherapy
Source: Front Oncol. 2021 Feb 11;10:610691. doi: 10.3389/fonc.2020.610691 (PMC7905101; doi:10.3389/fonc.2020.610691)
Supplement: Supplementary file 1 [file Table_1.docx]

**Supplementary table s1: List of texture features extracted in this study**

| Feature  class | Features description | Features | Number  of features |
| --- | --- | --- | --- |
| First Order | The distribution of voxel intensities within the image region defined by the mask through commonly used and basic metrics | 10Percentile,90Percentile,Energy,Entropy,InterquartileRange,Kurtosis,Maximum,MeanAbsoluteDeviation,Mean,Median,Minimum,Range,RobustMeanAbsoluteDeviation,RootMeanSquared,Skewness,TotalEnergy,Uniformity,Variance | 18 |
| Shape | Descriptors of the three-dimensional size and shape of the ROI | Elongation,Flatness,LeastAxis,MajorAxis,Maximum2DDiameter(Column),Maximum2DDiameter(Row),Maximum2DDiameterSlice,Maximum3DDiameter,MinorAxis,Sphericity,SurfaceArea,SurfaceVolumeRatio,Volume | 13 |
| GLCM | The second-order joint probability function of an image region constrained by the mask | Autocorrelation,JointAverage,ClusterProminence,ClusterShade,ClusterTendency,Contrast,Correlation,DifferenceAverage,DifferenceEntropy,,DifferenceVariance,JointEnergy,JointEntropy,Imc1,Imc2,Idm,Idmn,Id,Idn,InverseVariance,MaximumProbability,SumAverage,SumEntropy,SumSquares | 23 |
| GLSZM | Quantifies gray level zones in an image | GrayLevelNonUniformity,GrayLevelNonUniformityNormalized,GrayLevelVariance,HighGrayLevelZoneEmphasis,LargeAreaEmphasis,LargeAreaHighGrayLevelEmphasis,LargeAreaLowGrayLevelEmphasis,LowGrayLevelZoneEmphasis,SizeZoneNonUniformity,SizeZoneNonUniformityNormalized,SmallAreaEmphasis,SmallAreaHighGrayLevelEmphasis,SmallAreaLowGrayLevelEmphasis,ZoneEntropy,ZonePercentage,ZoneVariance | 16 |
| GLRLM | Quantifies gray level runs, which are defined as the length in number of pixels, of consecutive pixels that have the same gray level value | GrayLevelNonUniformity,GrayLevelNonUniformityNormalized,GrayLevelVariance,HighGrayLevelRunEmphasis,LongRunEmphasis,LongRunHighGrayLevelEmphasis,LongRunLowGrayLevelEmphasis,LowGrayLevelRunEmphasis,RunEntropy,RunLengthNonUniformity,RunLengthNonUniformityNormalized,RunPercentage,RunVariance,ShortRunEmphasis,ShortRunHighGrayLevelEmphasis,ShortRunLowGrayLevelEmphasis | 16 |
| NGTDM | Quantifies the difference between a gray value and the average gray value of its neighbours within distance δ | Busyness, Coarseness, Complexity, Contrast, Strength | 5 |
| GLDM | Quantifies gray level dependencies in an image | DependenceEntropy,DependenceNonUniformity,DependenceNonUniformityNormalized,DependenceVariance,GrayLevelNonUniformity,GrayLevelVariance,HighGrayLevelEmphasis,LargeDependenceEmphasis,LargeDependenceHighGrayLevelEmphasis,LargeDependenceLowGrayLevelEmphasis,LowGrayLevelEmphasis,SmallDependenceEmphasis,SmallDependenceHighGrayLevelEmphasis,SmallDependenceLowGrayLevelEmphasis | 14 |

**GLCM, gray-level co-occurrence matrix,; GLRLM, gray-level run-length matrix; GLSZM, gray-level size zone matrix; NGTDM, neighbourhood gray-tone difference matrix.**

**Note: it's worth noting that the names in this list are different from IBSI names, but the methods are the same. The algorithm details about feature extraction can be referred to:** [**https://pyradiomics.readthedocs.io**](https://pyradiomics.readthedocs.io)

**File 2**

Rad-score =firstorder_Energy * (-4.021055e-10) + firstorder_Maximum * (-7.998650e-05) + firstorder_Median * (1.796902e-03) + firstorder_Skewness * (-1.415817e-02) + glrlm_GrayLevelVariance * (-2.163660e-02) + glrlm_ShortRunLowGrayLevelEmphasis * (-9.746240e-01) + glszm_ZoneEntropy * (-4.959256e-02) + gldm_DependenceNonUniformityNormalized * (5.750932e+00) + gldm_SmallDependenceLowGrayLevelEmphasis * (-7.208450e+00) +

ngtdm_Strength * (-4.075127e-02)
